# "The Christian World" and Mr. Alabone

**Published:** 1910-07-30

**Authors:** 


### The Hospital

A Journal of

The Medical Sciences and Hospital Administration.

New Series. No. 181, Vol. VII. [No. 1253, Vol. XLVIII.] SATURDAY, JULY 30, 1910

### "THE CHRISTIAN WORLD" AND MR. ALABONE

There is a kind of polemic, common in minor forensic practice, which consists in assailing a position with any sort of specious argument, the question of inherent reasonableness being carefully ignored. When, in addition, it proceeds from a party pecuniarily interested, and almost of necessity personally embittered, it plainly belongs to that class of criticism which, in Huxley's phrase, one looks at and passes by. We should not, indeed, have troubled to notice a long letter of the *provenance* indicated above had it not been for the following editorial backing:—

"The Hospital, in its last issue, complains of the 'gross misstatements' made by Dr. Alabone on the 'open-air' cure for consumption. Without in any way denying the beneficial effect of constant contact with pure air, of which Dr. Alabone has always been a strong advocate, it seems to us that he has ample ground for his criticisms of the way in which patients are recklessly exposed to the elements regardless of their condition. He has numberless letters, many of which we have seen, from patients who have been at these institutions, and who have come away much worse for their treatment. And many cases have come under his notice of patients who have apparently greatly benefited and have been discharged as cured and have died shortly afterwards. Dr. Alabone himself may possibly have something to say on the matter" (*The Christian World*, June 16, 1910).

These remarks clearly do nothing to explain the remarkable inconsistence, which we originally pointed out, of our contemporary's reviewing columns (recommending a book which advocated the establishment of many additional sanatoriums) with its advertisement ones; an inconsistence which becomes more remarkable than ever. However, this by the way. What seems forced upon us now is to supply intelligent laymen with a few pertinent and unmutilated facts, amplifying the remarks which we have already made on the subject of sanatoriums in former issues, and more especially in certain paragraphs of "The Week's Work" in The Hospital of July 23. It is an interesting thing, for instance, that a laudatory article in one of the literary reviews by an ex-patient—not a medical man—of Dr. Walther's institution, so widely copied here, did more than anything else to hasten the composed of laymen, who may be trusted to lend introduction of sanatoriums into this country. Then there are many authorities almost exclusively a sympathetic ear to complaints by patients of undue hardships, who maintain sanatorium beds—bodies, continually growing in number, like the large municipalities, like Poor-law Guardians, like the affiliated trade-unions and friendly societies who support Benenden Sanatorium in Kent, or like the Charity Organisation Society. It is indeed to be wished that those who read Mr. Alabone's diatribe could visit Germany, a country with no lack of irregular practitioners to vaunt their wares, and inspect the three hundred beds at Beelitz, mainly paid for out of workmen's insurance money. How, in view of all this, is the assertion that patients themselves are pretty unanimous in condemning sanatoriums as worse than useless to be described as other than a gross misstatement? Take, again, the statistics of the Brompton Hospital for Consumption, which go back to the year 1852. The results for that year, and for 1862, 1872, 1882, and 1892 are all much the same. Only in 1902 is there a sudden improvement, an improvement which has been maintained since, just because the existing treatment was introduced about that time. Now it is certain that in the previous fifty years many new remedies had been tried by the physicians; why should any supposed bias on their part show itself only in the case of the open-air method?

Naturally, considering the psychology of the consumptive, there will be discontented patients, and certainly exaggerated claims have been made for sanatorium treatment. Only recently we ourselves pointed out the dogmatism of certain statements in the book praised by our contemporary, and which

Professor Pearson has since convicted of a statistical inaccuracy. But its main thesis, that sanatorium treatment is an advance on older methods, will not be questioned now by the majority of medical men. Some will question it, of course, just as some (far fewer, however) might be found to disagree with the verdict "inert" pronounced, after painstaking trial by a responsible committee, upon lachnanthes as a cure for consumption. But while of the former state of things a hint may be found in "The School for Scandal," Sir Peter Teazle observing dolefully that his friends wished him happiness with his young bride much as one wishes good health to a man in consumption, nowadays Trudeau, Dettweiler—may we add St. Clair Thomson?—and others are examples of those who recover themselves to put others in the way of doing the same thing. It is a significant fact, in this connection, that Koch, as one learnt recently, "was not at all afraid of tuberculosis."

